# A Horrific Case of Proptosis: Salvaging Vision in a Pediatric Patient With Rapidly Growing Orbital Rhabdomyosarcoma

**DOI:** 10.7759/cureus.91332

**Published:** 2025-08-31

**Authors:** Kunalini Anpalagan, Christine Ong, Siu Wan Foo, Ee Ling Ang, Mae-Lynn C Bastion

**Affiliations:** 1 Department of Ophthalmology, Faculty of Medicine, Universiti Kebangsaan Malaysia Medical Centre, Kuala Lumpur, MYS; 2 Department of Ophthalmology, Hospital Canselor Tuanku Muhriz, Kuala Lumpur, MYS; 3 Department of Ophthalmology, Hospital Pulau Pinang, Pulau Pinang, MYS

**Keywords:** eye lid swelling, non-axial proptosis, optic nerve involvement, orbital rhabdomyosarcoma, paediatric oncology

## Abstract

We report the case of a four-year-old male who presented with painless right upper eyelid swelling initially treated as a chalazion. The swelling rapidly progressed within two weeks, resulting in non-axial proptosis, thereby restricting ocular motility and causing vision loss. Imaging revealed a large superomedial orbital mass with optic nerve displacement. Histopathology confirmed embryonal rhabdomyosarcoma (RMS) group III. This report highlights the importance of prompt biopsy, multidisciplinary care, and a multimodal approach in achieving favorable outcomes through early diagnosis and intervention despite rapid tumor growth and optic nerve involvement.

## Introduction

Rhabdomyosarcoma (RMS) is a rare and aggressive soft tissue tumor, accounting for 3% of childhood cancers and 2% of adolescent cancers; however, it is rare in adults [1,2,3]. It most commonly affects children aged one to four years (35%), followed by those aged 10-14 years (20%) and over 15 years (13%) [4]. There is a 1.5 times higher incidence in males compared to females [4]. The tumor is typically unilateral and shows no racial predisposition [4]. The head and neck (45%), trunk (40%), and extremities (15%) are the most common sites [4]. RMS manifests in various subtypes, including embryonal (about 60% of cases), alveolar (about 20%), pleomorphic (about 10%), and spindle/sclerosis (about 10%) [2]. Embryonal RMS has the best prognosis [4]. Orbital RMS represents approximately 10% of all RMS cases, making it a notable yet uncommon presentation [5]. Orbital RMS often presents as a rapidly enlarging, painless, unilateral proptosis, sometimes accompanied by eyelid swelling, ptosis, or restricted ocular motility. Due to its superficial location and overlap with benign eyelid pathologies such as chalazion or cellulitis, early diagnosis can be challenging, especially in primary care settings. Managing RMS is also complex due to its rapid growth and metastatic potential. Standard treatment typically involves a multimodal approach, including chemotherapy, surgical resection, and radiation therapy, tailored according to tumor subtype, site, and stage. We present a case of right orbital embryonal RMS in a pediatric patient.

## Case presentation

The patient was a four-year-old male with no comorbidities and no family history of malignancy who presented with a painless swelling on his right upper eyelid, which had been increasing in size for two weeks. He had been initially diagnosed with a chalazion by a general practitioner and treated conservatively with systemic and topical antibiotics one week ago; however, the swelling had continued to increase. The patient appeared well-nourished with a normal body build and weight-for-age, with no signs of malnutrition noted clinically.

Ocular examination revealed a firm, non-tender, approximately 2 cm mass on the right upper eyelid, which was fixed to the underlying tissue and not mobile with palpation. The patient had non-axial proptosis, and the overlying skin was mildly erythematous. The right eye's unaided visual acuity was 6/60 with a chin-up position, and that of the left eye was 6/9, with a negative relative afferent pupillary defect (RAPD). The right eye conjunctiva was injected and chemotic at the superonasal region; otherwise, the cornea was clear, and the anterior chamber was deep and quiet. The optic disc was not swollen, and the fundus was normal. The right eye had limited ocular movements in superior and medial gazes. Left eye anterior and posterior findings were unremarkable. Systemic examination revealed unremarkable findings and no palpable lymphadenopathy.

**Figure 1:**
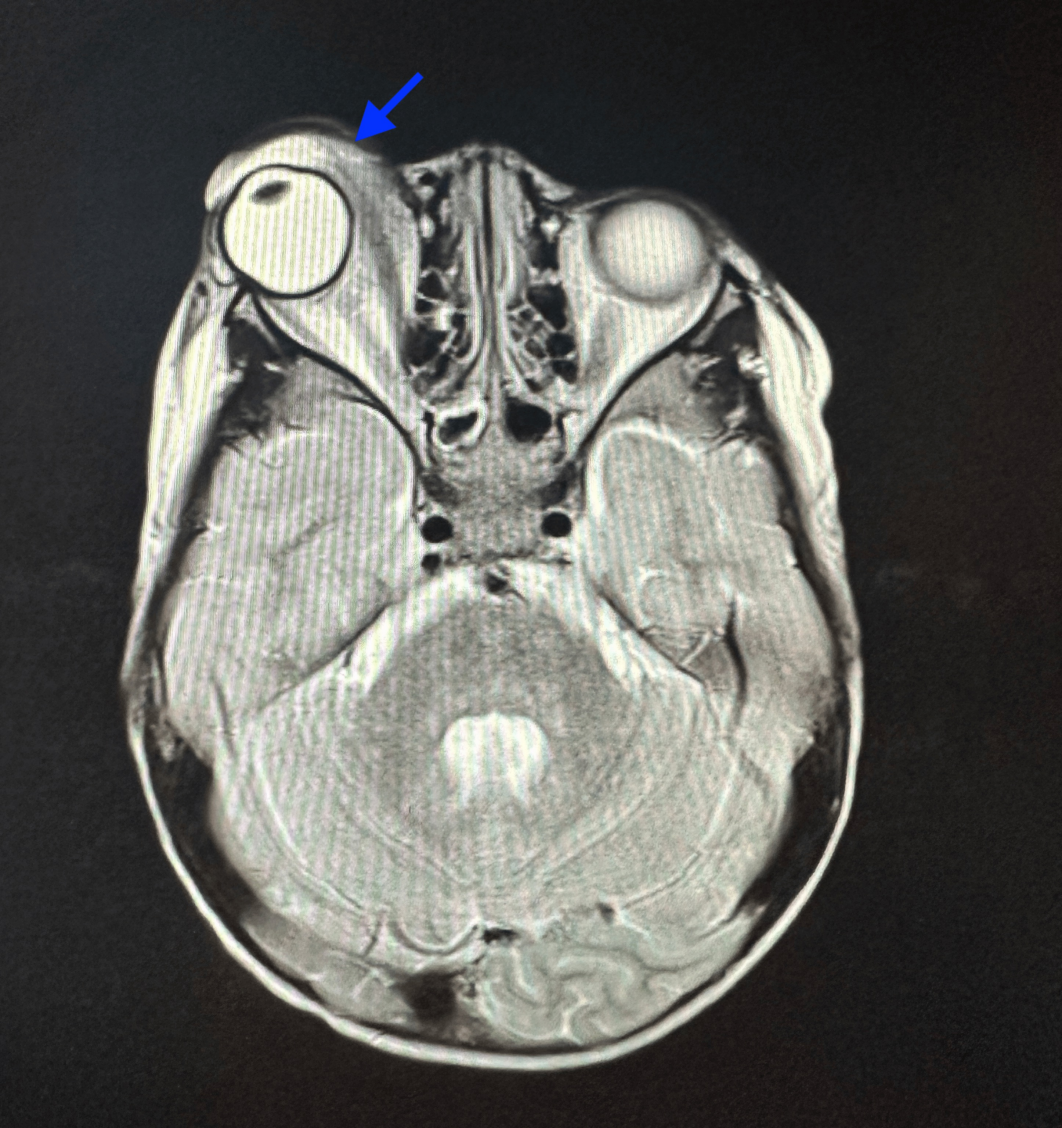
Axial T2-weighted MRI scan showing a heterogenous lobulated solid mass at superomedial aspect of the right orbit, displacing the globe inferolaterally (blue arrow) MRI: magnetic resonance imaging

**Figure 2:**
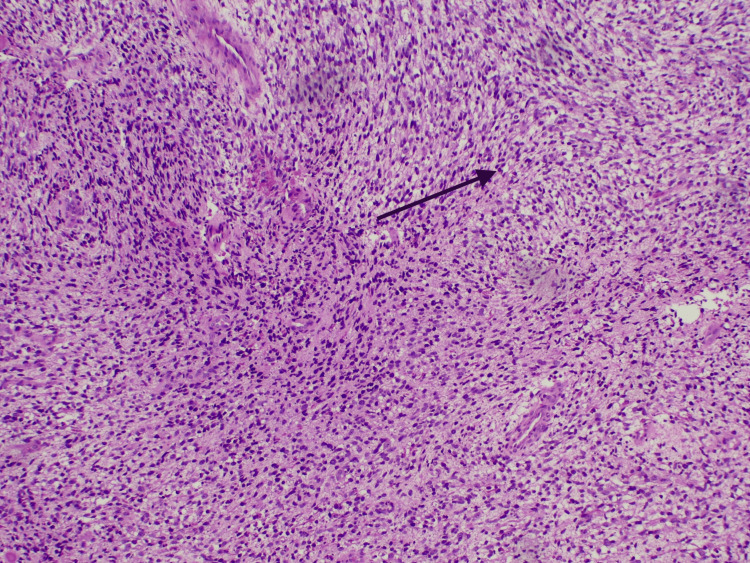
Hematoxylin and eosin stain of tumor cell shows pleomorphic spindle, stellate, and primitive-looking round cells (black arrow) These findings raised suspicion for rhabdomyosarcoma, prompting further immunohistochemical analysis

**Figure 3:**
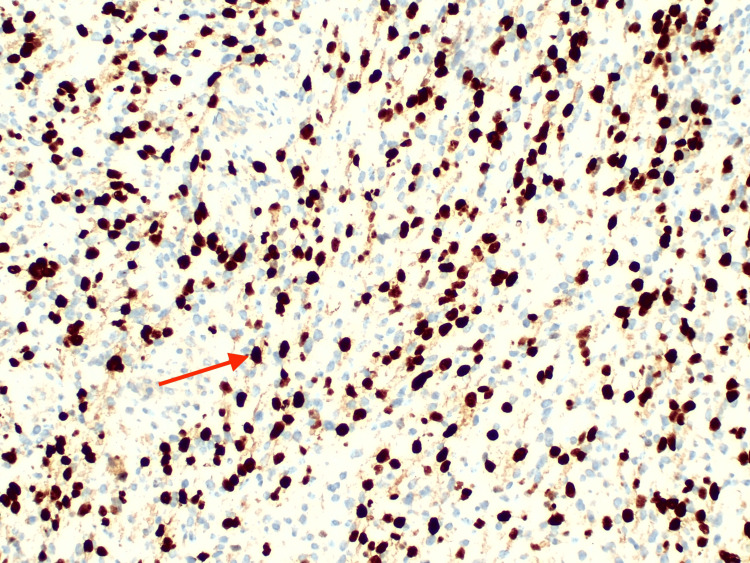
Tumor cells demonstrating strong positivity for desmin stain (red arrow) Desmin positivity supports myogenic differentiation and is a key marker for confirming the diagnosis of rhabdomyosarcoma, distinguishing it from other small round blue cell tumors in children

MRI of the brain and orbit confirmed the presence of a soft tissue mass at the superomedial aspect of the right orbit with no evidence of bony erosion or intracranial extension, measuring 3.5 x 2.8 x 2.4 cm (anteroposterior x width x craniocaudal) (Figure 1). Subsequently, the child was referred to the oculoplastic team and underwent an incisional biopsy, revealing embryonal RMS (Figures 2, 3). The child was co-managed with the pediatric oncology team. Staging investigations, including CT thorax and a bone scan, showed no lymph node involvement or metastasis, leading to the diagnosis of right orbital rhabdomyosarcoma, Intergroup Rhabdomyosarcoma Study (IRS) Group III pT3bN0M0 of embryonal subtype. Laboratory investigation was essentially normal with the exception of an increased platelet count (Table 1).

**Table 1:** Summary of laboratory investigations CRP: C-reactive protein; ESR: erythrocyte sedimentation rate

Variable	Result	Unit	Reference range
Haemoglobin	11.9	g/dL	11.0-14.0
White blood cell	13.2	10^3^/uL	5-15
Platelet	659	10^3^/uL	200-450
Urea	6.21	mmol/L	2.76-8.07
Creatinine	20	umol/L	27-42
Sodium	138	mmol/L	136-145
Potassium	4.1	mmol/L	3.4-4.5
CRP	<0.6	mg/L	<5.0
ESR	32	mm/hr	0-35

**Figure 4:**
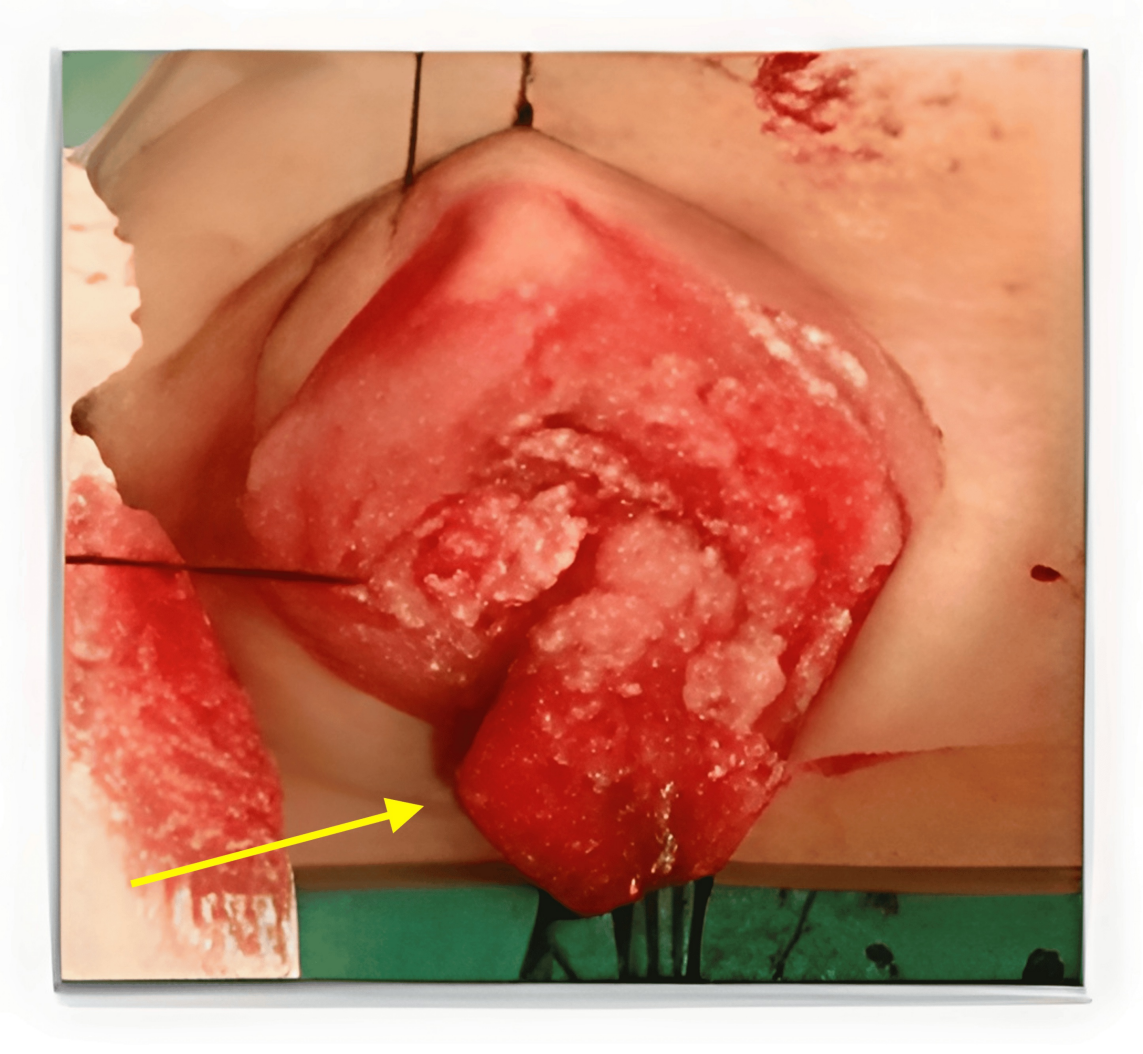
Tumor during the debulking procedure (yellow arrow)

**Figure 5:**
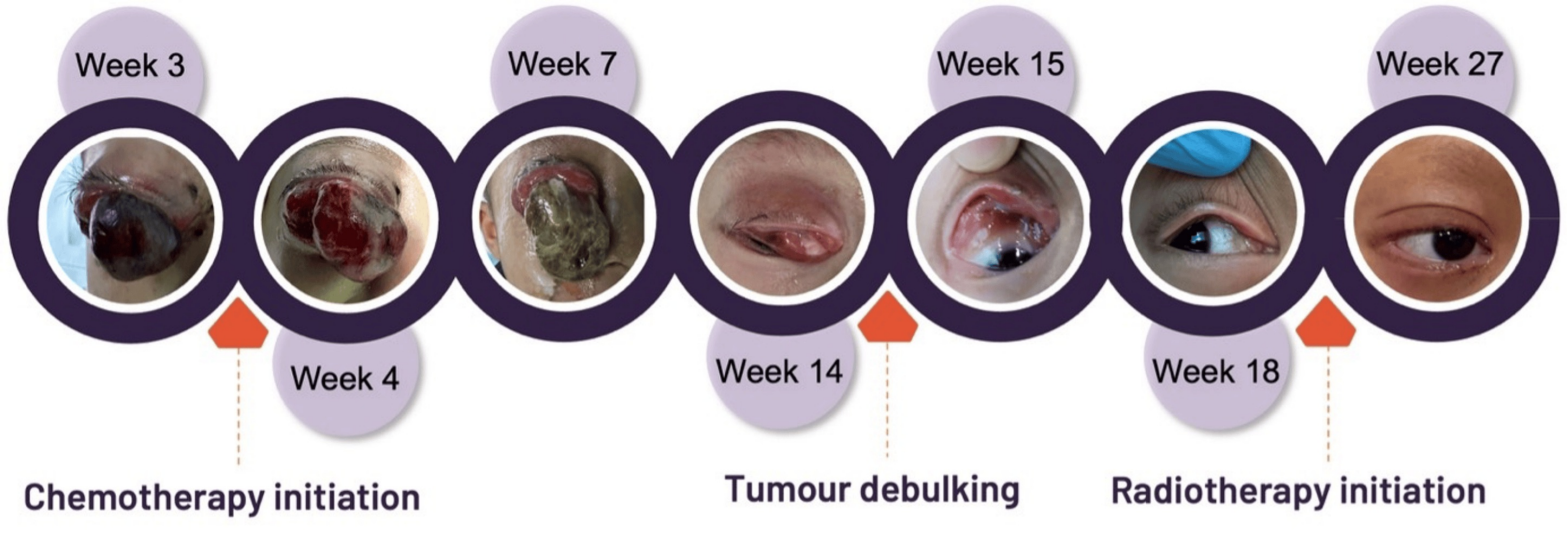
Chronological progression of photos from initial presentation to the completion of treatment

The patient underwent chemotherapy consisting of vincristine, actinomycin-D, and ifosfamide (IVA) based on the RMS 2005 Protocol Subgroup C developed by the European Pediatric Soft Tissue Sarcoma Study Group (EpSSG). At week seven of illness onset, the tumor grew in size, and a positive RAPD developed. However, following three cycles of chemotherapy, MRI showed that the tumor had reduced to 1.4 x 1.7 x 1.3 cm. Tumor debulking surgery was performed (Figure 4), followed by five additional cycles of chemotherapy and concomitant radiotherapy of 45 Gray in 25 fractions at the tumor site, which reduced the tumor further to 0.4 x 0.9 x 0.1 cm (Figure 5).

Postoperative VA was 1/60, improving to 6/48 after two months of amblyopia therapy with daily fellow eye patching; however, it reduced again to 2/60 due to radiation-related keratopathy. Six months after completing chemotherapy and radiotherapy, VA improved to 6/12 following intensive treatment for amblyopia and keratopathy. Repeated MRI showed that the tumor size remained at 0.4 x 0.7 x 0.1 cm. The patient is currently under three-monthly follow-up for repeat MRI scans.

## Discussion

Orbital RMS cases, while well-documented in literature globally, remain exceedingly rare in our region, as reported by Qi-Xian et al.; no cases were found in a five-year local case series (2016-2020) [6]. This rarity contributes to its misdiagnosis, as seen in our case, where the initial presentation mimicked a benign chalazion. The differential diagnoses of orbital RMS are broad, including orbital cellulitis, idiopathic orbital inflammation, dermoid cyst, Langerhans cell histiocytosis (eosinophilic granuloma), haemangioma, myeloid sarcoma, lymphangioma, metastatic neuroblastoma, and lymphoma. Distinguishing RMS from these conditions is critical for appropriate and timely management to avoid delays that may compromise both vision and survival.

Management of this case was guided by the IRS Group guidelines, which remain a cornerstone for risk stratification and treatment planning in pediatric RMS [5]. The initial step is a tissue biopsy to obtain a histologic diagnosis [7]. In our case, the aggressive nature of the tumor became apparent during early chemotherapy, with rapid growth and new-onset RAPD indicating optic nerve compromise. This emphasizes that early biopsy is not just a diagnostic formality but a time-critical step in initiating life and sight-saving therapy. Excisional biopsy is ideal when feasible, as it removes the tumor and confirms the diagnosis, which is significant for a better outcome [8]. However, it was not possible here due to the tumor size and proximity to vital structures. Incisional biopsy thus served as a practical and effective alternative.

The initial tumor enlargement observed following chemotherapy may be attributed to tumor necrosis and inflammatory response, which can cause temporary swelling of the lesion. This phenomenon, sometimes referred to as “tumor flare”, is not uncommon in RMS and may mimic disease progression, especially during the first few treatment cycles. Another possibility is rapid tumor cell turnover, leading to a transient increase in mass effect before subsequent reduction in tumor volume. Awareness of this expected response is important to avoid premature changes in the treatment plan.

Multimodal treatment remains the gold standard, integrating surgery, multidrug chemotherapy, and radiotherapy [9]. Imaging plays a pivotal role at every stage; CT or MRI scans are used to assess the tumor size, examine nearby lymph nodes, and detect distant metastases [7]. Specifically, CT helps in detecting bony involvement, while MRI provides detailed soft tissue contrast and information about intracranial spread [1]. In our case, MRI was the preferred modality for follow-ups due to the absence of bone invasion and its utility in monitoring residual disease.

Although the embryonal subtype of RMS carries a favorable prognosis, early diagnosis and intervention are crucial, particularly when optic nerve compression occurs early. Our case highlights the paradox that even tumors may exhibit rapid growth during the initial phase of chemotherapy. This pattern has been previously reported, such as the case by Chitsike et al., where an apparent early response was followed by local progression during the third cycle of chemotherapy [10]. In addition, the timely introduction of radiotherapy of 45 Gray was crucial in achieving local tumor control in our case. This aligns with the findings of Cassady et al., who reported high success rates with similar dosing in patients with microscopic residual disease [11].

Survival outcomes in orbital RMS are excellent, with five-year survival rates exceeding 90% in multiple series, such as those reported by Wang et al. and Shields et al. [1,5]. However, Shields et al. have shown that long-term visual outcomes are more variable; 20/40 or better in 39% while 20/200 or worse in 43% [5]. During the initial phase of chemotherapy, the tumor increased in size and a positive RAPD was observed, suggesting optic nerve compression as a contributing factor to vision loss. Also, the patient had maintained poor vision since presentation despite a structurally normal retina and optic disc, and in the absence of prior visual impairment or refractive error, suggesting stimulus deprivation amblyopia due to the orbital mass. In our patient, visual recovery from 6/60 to 6/12 was achieved through timely tumor control, amblyopia therapy, and management of radiation-induced keratopathy. Post-treatment follow-ups are essential to monitor recurrence, secondary tumors, and late radiotherapy side effects [7]. Ocular examinations and MRI should be conducted at three-month intervals, reducing in frequency over five years post-treatment [5].

## Conclusions

This report highlights the value of early biopsy, timely radiotherapy, and multidisciplinary care in managing pediatric orbital RMS. Prompt diagnosis and multimodal treatment led to a favorable functional outcome in our patient, with vision improving to 6/12. It reinforces the need for maintaining a high index of suspicion when encountering cases of atypical eyelid swellings and throws light on the strategies to preserve vision while achieving tumor control.
